# Association between dietary calcium intake and constipation in a metabolic syndrome population: evidence from NHANES 2005–2010

**DOI:** 10.3389/fnut.2024.1422564

**Published:** 2024-10-30

**Authors:** Li Zhu, Long Yang, Zonghua Liang, Wen Shi, Ming Ma, Jingbo Chen, Zulipikaer Abdula, Xuchen Gong

**Affiliations:** ^1^Department of Anus and Intestine Surgery, People’s Hospital of Xinjiang Uygur Autonomous Region, Xinjiang Uygur Autonomous Region, Urumqi, China; ^2^Pediatric Cardiothoracic Surgery, First Affiliated Hospital of Xinjiang Medical University, Urumqi, China; ^3^Research and Education Center, People’s Hospital of Xinjiang Uygur Autonomous Region, Urumqi, China; ^4^Department of Traditional Chinese Medicine, People’s Hospital of Xinjiang Uygur Autonomous Region, Xinjiang Uygur Autonomous Region, Urumqi, China

**Keywords:** metabolic syndrome, calcium, dietary, constipation, NHANES

## Abstract

**Background:**

The global prevalence of Metabolic Syndrome (MetS) is increasing, primarily characterized by abdominal obesity, which significantly heightens the risk of cardiovascular diseases, gastrointestinal disorders, and cancers. Constipation is a common gastrointestinal issue that impacts both physiological and psychological health and worsens with age. Calcium, an essential mineral vital for human health, has been proven to be crucial not only for bone health but also beneficial for gastrointestinal health. However, the results regarding its impact on constipation are inconsistent. This study aimed to investigate the relationship between dietary calcium intake and constipation in individuals with MetS.

**Methods:**

This cross-sectional study utilized data from the National Health and Nutrition Examination Survey (NHANES) from 2005 to 2010. Participants were assessed for MetS based on the International Diabetes Federation (IDF) criteria. Dietary calcium intake was evaluated through 24-h dietary recalls, and constipation was defined based on the frequency of bowel movements recorded in the bowel health questionnaire. The relationship between calcium intake and constipation was explored using logistic regression models with adjustment for covariates, and restricted cubic spline analyses were also used to investigate nonlinear relationships.

**Results:**

The study included 4,838 adult participants with MetS. Adjusted logistic regression revealed that an increase in dietary calcium intake was significantly associated with a reduced risk of constipation (OR: 0.562, 95% CI: 0.379 to 0.835, *p* = 0.006). Compared to the lowest quartile, the highest quartile of dietary calcium intake significantly decreased the risk of constipation (OR: 0.282, 95% CI: 0.115 to 0.691, *p* = 0.008). Results from the restrictive cubic spline analysis indicated a negative linear association between dietary calcium intake and constipation risk (non-linearity *p* = 0.704).

**Conclusion:**

The findings suggested that increased dietary calcium intake is associated with a decreased risk of constipation among MetS patients, emphasizing dietary calcium as a potentially modifiable factor for managing gastrointestinal symptoms in this population.

## Backgrounds

With changes in modern societal lifestyles, the prevalence of Metabolic Syndrome (MetS) has been gradually increasing worldwide (1). MetS, characterized primarily by abdominal obesity, substantially raises the risk of cardiovascular diseases, gastrointestinal disorders, and cancers (2). It is estimated that about one-quarter of the global population bears this disease burden (3). The high prevalence of MetS poses a significant challenge to public health systems, increasing the burden of heart disease, diabetes, and other non-communicable diseases, and leading to a marked increase in healthcare costs (4).

Constipation is a common gastrointestinal disorder, defined by the World Gastroenterology Organisation (WGO) as a decrease in bowel movement frequency (fewer than three times per week), difficulty in defecation, or a sensation of incomplete evacuation (5). Constipation not only affects physiological health but also harms psychological and social functions, thereby reducing quality of life (6, 7). It is estimated that between 10.1 to 15.3% of adults globally suffer from chronic constipation, with a higher prevalence in females than males, and an increasing rate with age (8, 9). Furthermore, studies have shown that long-term constipation significantly increases the risk of gastric cancer, colorectal cancer, and other digestive system cancers (10, 11). Obesity is related to constipation, with risk factors including, but not limited to, dietary habits, reduced physical activity, and changes in intestinal hormones (12, 13). Therefore, timely management and intervention for the MetS population, primarily characterized by abdominal obesity, are crucial for preventing various gastrointestinal diseases.

In recent years, dietary factors have received increasing attention in the prevention and management of constipation. Calcium, an essential mineral for maintaining human health, has been proven to be critical not only for bone health but also for gastrointestinal health (14). Several studies suggest that increasing dietary calcium intake may reduce the risk of colorectal cancer (15–17). Additionally, patients with irritable bowel syndrome (IBS) have been shown to have significantly lower dietary calcium intake compared to the general population (18). Calcium plays a role in preventing and alleviating constipation and other digestive disorders by promoting intestinal motility and maintaining the stability of the intestinal microenvironment. Most studies related to constipation have focused on the use of calcium supplements or calcium-based medications. For instance, Alyousif et al. found that calcium supplementation was not associated with constipation in healthy women (19). On the other hand, Lewis et al. reported that the use of calcium supplements in myocardial infarction patients was often linked to gastrointestinal symptoms, particularly constipation (20). However, research directly investigating the relationship between dietary calcium intake and constipation remains limited. Therefore, this study aims to explore the association between dietary calcium intake and constipation among MetS patients, using data from the National Health and Nutrition Examination Survey (NHANES) from 2005 to 2010.

## Methods

### Study design and population

**Figure 1:**
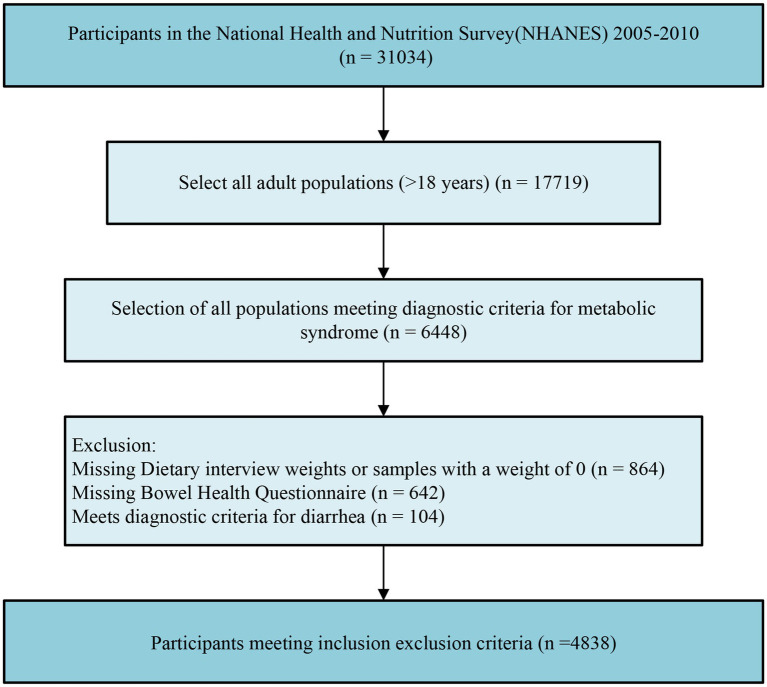
Flow chart of the study design.

This study is a cross-sectional analysis using data from the National Health and Nutrition Examination Survey (NHANES) database from 2005 to 2010. The aim is to investigate the relationship between daily dietary calcium intake and the prevalence of constipation among individuals with MetS. NHANES is a research program conducted by the National Center for Health Statistics (NCHS), a division of the Centers for Disease Control and Prevention (CDC), designed to assess the health and nutritional status of adults and children in the United States (21). In this study, we initially collected data on 31,034 participants from NHANES 2005–2010 and excluded those under the age of 18. Additionally, an evaluation was conducted to determine the presence of MetS among all participants, selecting those within the MetS population, and further excluding individuals with missing sample weights, missing bowel health questionnaires, and those diagnosed with diarrhea. After applying these criteria, the study included a total of 4,838 individuals with MetS (Figure 1). The study’s data collection was approved by the Research Ethics Review Committee of the National Center for Health Statistics, and all participants provided written informed consent.

### Data collection

Initially, we obtained information on participants’ gender, age, education level, race, and marital status from the “Demographics Data.” We also collected blood pressure, weight, height, and waist circumference data from the “Examination Data.” From the “Laboratory Data,” we gathered metrics such as triglycerides (TG), lymphocytes, neutrophils, platelets, total cholesterol (TC), high-density lipoprotein cholesterol (HDL-C), low-density lipoprotein cholesterol (LDL-C), C-reactive protein(CRP), serum uric acid, serum creatinine, and fasting glucose levels. The “Questionnaire Data” allowed us to assess participants’ specific disease information, including physical activity, cardiovascular diseases (CVD), alcohol consumption, smoking, hypertension, diabetes, and bowel conditions.

Participants were categorized into young men (age < 40), middle-aged men (40 ≤ age < 60), older men (age ≥ 60), premenopausal women (age < 50), and postmenopausal women (age ≥ 50) based on their age and gender. Smoking status was defined based on the quantity and duration of smoking throughout a lifetime: “never” (fewer than 100 cigarettes smoked), “former” (over 100 cigarettes smoked in a lifetime but currently not smoking at all), and “current” (over 100 cigarettes smoked in a lifetime and currently smoking some days or every day). Participants were categorized into “light,” “moderate,” “heavy,” and “never” drinkers based on the amount of alcohol consumed daily over a year. A “light” drinker was defined as a female who consumes up to 1 drink per day and a male who consumes up to 2 drinks per day within a year; a “moderate” drinker was defined as a female who consumes up to 2 drinks per day and a male who consumes up to 3 drinks per day within a year; a “heavy” drinker was defined as a female who consumes 3 or more drinks per day and a male who consumes 4 or more drinks per day within a year. Physical activity was assessed based on Metabolic Equivalent of Task (MET) values, categorized as optimal (≥8,000 MET-minutes/week), moderate (600–7,999 MET-minutes/week), and poor (<600 MET-minutes/week) (22). Calculation of the systemic immune-inflammatory index (SII) using three indices: lymphocytes, neutrophils, and platelets (23). Cardiovascular disease (CVD) was identified if participants affirmatively answered that they had been diagnosed by a doctor with coronary heart disease, angina, heart attack, stroke, or atrial fibrillation. Chronic kidney disease (CKD) was defined as having a glomerular filtration rate (eGFR) < 60 mL/min/1.73 m^2, or a urine albumin-to-creatinine ratio ≥ 25 mg/g for women and ≥ 17 mg/g for men. A diagnosis of chronic renal failure was considered if any of the aforementioned conditions were met (24). All of the above information can be downloaded from the NHANES website under “Survey Data and Documentation.”

### Dietary intake assessment

Dietary consumption data were collected from 24-h recalls (midnight to midnight) prior to the interview. The data collection for the dietary interview section was conducted by the National Center for Health Statistics (NCHS) of the Department of Health and Human Services (DHHS), under the oversight of the U.S. Department of Agriculture (USDA). The USDA’s Food Surveys Research Group (FSRG) is responsible for the methodology of dietary data collection, maintenance of the database used for coding and processing the data, and the auditing and processing of the data (25, 26). An effective assessment was then made of the types and quantities of foods and beverages consumed, as well as the quantities of nutrients and other food components, including total energy, carbohydrates, total saturated fatty acids (TFA), fiber, protein, and calcium. For our study, we utilized the dietary recall data from the first 24-h interview.

### Diagnostic criteria for metabolic syndrome

In our study, the diagnosis of MetS was based on the International Diabetes Federation (IDF) criteria established in 2009, which emphasizes central obesity as a mandatory criterion, along with the presence of two or more additional metabolic factors (27). For central obesity, waist circumference thresholds are set at ≥94 cm for men and ≥ 80 cm for women, adjusted for the American population. Additional factors include raised triglycerides (≥150 mg/dL), reduced HDL cholesterol (<40 mg/dL in males and < 50 mg/dL in females), elevated blood pressure (≥130/85 mm Hg), or elevated fasting glucose (≥100 mg/dL), or treatment for these conditions.

### Definition of constipation

In NHANES, constipation was assessed using defecation frequency and stool consistency, as recorded by participants in the bowel health questionnaire. The determination of constipation relied primarily on defecation frequency, given the weak correlation observed between stool frequency and consistency (28). During the data collection phase, participants reported their average weekly bowel movements. Based on this information, defecation frequencies of fewer than three times per week were categorized as constipation, frequencies between three and 21 times per week were considered normal, and frequencies exceeding 21 times per week were identified as diarrhea, consistent with prior NHANES findings (29, 30).

### Statistical analysis

All analyses in this study were conducted using R software (version 4.3.1). Initially, participants were divided into quartiles based on their dietary calcium intake, and their baseline characteristics were compared. Categorical variables were represented by frequencies and weighted percentages, with intergroup comparisons made using the Rao-Scott chi-square test. Continuous variables were expressed using weighted means and standard deviations, and comparisons among groups were performed using one-way analysis of variance (ANOVA). Furthermore, logistic regression models were employed to investigate the relationship between dietary calcium intake and the incidence of constipation, adjusting for a range of covariates such as gender, age, race, specific laboratory indicators, and other nutrients. Additionally, stratified analyses were conducted to carefully examine the associations within different demographic characteristics, including age groups, gender, and components of MetS. Finally, restricted cubic splines (RCS) were used to explore the nonlinear associations between dietary calcium intake and the occurrence of constipation. All analyses accounted for the inherent stratified and clustered sampling design of NHANES and were weighted accordingly, with a two-sided *p*-value <0.05 considered statistically significant (31, 32).

## Results

### Baseline characteristics of MetS populations grouped by dietary calcium intake quartiles

In this study, we included 4,838 adult participants with MetS. The mean age of the cohort was 53.6 years, comprising 2,383 males (48.8%) and 2,455 females (51.2%), with 154 individuals (3.4%) suffering from constipation. Participants were further divided into four groups based on quartiles of dietary calcium intake: Q1 (<502.25 mg), Q2 (502.25 mg to 766 mg), Q3 (766 mg to 1113.75 mg), and Q4 (>1113.75 mg). Analysis revealed distinct characteristics across the calcium intake quartiles (Table 1). In the lowest calcium intake group (Q1), participants were older with an average age of 56.3 years, and had higher proportions of females, current smokers, and prevalence of CVD and CKD, along with lower levels of education and optimal physical activity; moreover, this group had lower waist circumference, albumin levels, dietary energy, protein, carbohydrate, and fiber levels, and higher C-reactive protein levels (all *p* < 0.05). Notably, the highest number of constipation cases was observed in Q1, with 56 individuals (5.6%). As dietary calcium intake increased from Q1 to Q4, there was a trend toward younger age, lower proportions of females, current smokers, CVD, CKD, and a lower prevalence of constipation. In the highest calcium intake group (Q4), the average age was 50 years with a higher proportion of males, 752 (61.1%). Additionally, this group had lower proportions of current smokers, CVD, CKD, and the highest level of optimal physical activity. Furthermore, participants in Q4 had higher waist circumference, albumin, dietary energy, protein, carbohydrate, and fiber levels. In terms of MetS components, quartile 4 exhibited lower prevalence of diabetes and hypertension. Remarkably, the lowest prevalence of constipation was observed in this quartile, with 23 cases (1.3%).

**Table 1:** Baseline characteristics of the metabolic syndrome population overall and grouped according to dietary calcium intake quartiles.

Characteristics	Overall	Q1 (< 502.25 mg)	Q2 (502.25 mg to 766 mg)	Q3 (766 mg to 1113.75 mg)	Q4 (> 1113.75 mg)	*p*- value
	*N* = 4,838	*N* = 1,210	*N* = 1,214	*N* = 1,204	*N* = 1,210	
Demographic information						
Age, year	53.6 ± 0.5	56.3 ± 0.7	54.6 ± 0.8	54.2 ± 0.7	50.0 ± 0.6	<0.001
Sex						<0.001
Female	2,455 (51.2)	730 (63.9)	661 (56.1)	594 (48.5)	470 (38.9)	
Male	2,383 (48.8)	483 (36.1)	553 (43.9)	621 (51.5)	726 (61.1)	
Ethnic						<0.001
Non-Hispanic White	2,576 (74.3)	554 (66.1)	633 (73.6)	682 (77.0)	707 (78.7)	
Non-Hispanic Black	808 (9.2)	304 (15.2)	213 (9.6)	166 (7.8)	125 (5.4)	
Mexican American	896 (8.6)	209 (8.4)	224 (9.3)	227 (7.3)	236 (9.5)	
Other race	558 (7.9)	146 (10.3)	144 (7.5)	140 (7.9)	128 (6.4)	
Marital						0.003
Never married	431 (9.6)	104 (8.3)	116 (10.0)	96 (7.6)	115 (12.6)	
With-partner	3,064 (67.0)	725 (64.8)	755 (66.4)	779 (67.1)	805 (69.1)	
Without-partner	1,343 (23.4)	384 (26.9)	343 (23.6)	340 (25.3)	276 (18.4)	
Education						<0.001
Junior high school and below	692 (7.6)	219 (11.4)	179 (8.8)	149 (5.4)	145 (5.8)	
High school and above	4,146 (92.4)	994 (88.6)	1,035 (91.2)	1,066 (94.6)	1,051 (94.2)	
Smoking						0.025
Never	2,348 (49.2)	555 (45.0)	586 (47.7)	604 (50.5)	603 (52.8)	
Former	1,532 (30.2)	381 (29.5)	403 (32.3)	402 (32.6)	346 (26.6)	
Now	958 (20.6)	277 (25.5)	225 (20.1)	209 (16.9)	247 (20.7)	
Drinking						<0.001
Never	735 (12.1)	242 (16.6)	185 (11.8)	169 (10.9)	139 (10.1)	
Former	1,286 (23.3)	366 (28.0)	323 (24.1)	313 (21.9)	284 (20.2)	
Mild	1,492 (34.6)	305 (25.8)	379 (36.1)	416 (36.0)	392 (38.8)	
Moderate or heavy	1,325 (30.0)	300 (29.6)	327 (28.0)	317 (31.1)	381 (30.9)	
Physical activity						0.009
Poor	1,636 (35.0)	440 (36.6)	435 (37.5)	407 (36.9)	354 (29.5)	
Intermediate	2,666 (54.4)	664 (54.4)	645 (53.5)	675 (53.0)	682 (56.8)	
Ideal	536 (10.6)	109 (9.0)	134 (9.0)	133 (10.1)	160 (13.7)	
CVD	971 (16.2)	301 (19.6)	255 (17.5)	233 (16.5)	182 (11.9)	0.003
CKD	1,396 (23.2)	430 (31.8)	340 (22.1)	341 (22.9)	285 (17.6)	<0.001
Physical and laboratory examinations						
BMI, kg/m^2^	32.2 ± 0.2	31.8 ± 0.3	32.1 ± 0.2	32.4 ± 0.4	32.3 ± 0.3	0.514
Waist cm	108.1 ± 0.4	106.7 ± 0.6	107.2 ± 0.4	108.9 ± 0.8	109.2 ± 0.7	0.010
CRP, mg/dL	0.50 ± 0.02	0.61 ± 0.04	0.49 ± 0.03	0.52 ± 0.04	0.41 ± 0.02	<0.001
SII	603.1 ± 8.4	600.4 ± 14.6	600.6 ± 12.0	622.2 ± 22.4	588.8 ± 17.0	0.686
Glucose, mmol/L	6.37 ± 0.04	6.48 ± 0.09	6.39 ± 0.06	6.40 ± 0.09	6.23 ± 0.08	0.141
Albumin, g/L	42.11 ± 0.08	41.47 ± 0.14	42.31 ± 0.13	42.19 ± 0.13	42.37 ± 0.13	<0.001
Creatinine, μmol/l	83.62 ± 0.77	85.06 ± 1.47	82.32 ± 0.87	83.24 ± 1.09	84.05 ± 1.60	0.366
Uric acid, μmol/L	351.54 ± 1.97	352.53 ± 3.11	350.15 ± 3.15	354.45 ± 3.45	349.05 ± 3.84	0.574
Triglyceride, mmol/L	2.85 ± 0.05	2.71 ± 0.10	2.90 ± 0.12	2.81 ± 0.11	2.95 ± 0.09	0.253
TC, mmol/L	5.22 ± 0.03	5.26 ± 0.05	5.27 ± 0.05	5.21 ± 0.05	5.17 ± 0.05	0.580
HDL, mmol/L	1.15 ± 0.01	1.18 ± 0.02	1.17 ± 0.01	1.15 ± 0.01	1.10 ± 0.01	0.004
LDL, mmol/L	2.80 ± 0.02	2.86 ± 0.04	2.79 ± 0.04	2.81 ± 0.04	2.74 ± 0.05	0.170
Dietary intakes						
Energy, kcal	2066.51 ± 24.71	1386.02 ± 27.45	1827.88 ± 27.20	2173.90 ± 37.10	2729.75 ± 46.76	<0.001
TFA, gm	26.5 ± 0.5	15.5 ± 0.4	22.1 ± 0.4	27.4 ± 0.6	38.2 ± 1.0	<0.001
Protein, gm	80.83 ± 1.12	53.62 ± 1.33	69.87 ± 1.31	84.79 ± 1.37	108.98 ± 2.06	<0.001
Carbohydrate, gm	249.44 ± 2.83	168.22 ± 3.54	222.36 ± 4.87	263.81 ± 4.33	325.76 ± 5.48	<0.001
Fiber, gm	15.98 ± 0.26	10.32 ± 0.29	14.43 ± 0.28	17.24 ± 0.43	20.74 ± 0.49	<0.001
MetS components						
MetS.Diabetes	2,896 (52.3)	779 (58.4)	732 (51.4)	732 (53.9)	653 (46.5)	0.003
MetS.low-HDL-C	2,805 (61.8)	709 (63.6)	693 (60.6)	688 (59.1)	715 (64.2)	0.328
MetS.TG	3,333 (73.2)	784 (69.3)	838 (73.0)	850 (75.3)	861 (74.4)	0.157
MetS.Obesity	4,706 (97.6)	1,175 (97.5)	1,178 (97.8)	1,192 (98.5)	1,161 (96.7)	0.227
MetS.Hypertension	3,310 (65.2)	884 (68.4)	849 (66.2)	838 (67.9)	739 (59.0)	0.010
Constipation	154 (3.4)	56 (5.6)	34 (3.7)	41 (3.5)	23 (1.3)	0.001

Continuous variables are expressed as weighted mean ± standard deviation and categorical variables are expressed as frequencies (weighted percentages). BMI, body mass index; CRP, C reactive protein; SII, systemic immune-inflammation index; TC, total Cholesterol; HDL, high-density lipoprotein; LDL, low-density lipoprotein; CVD, cardiovascular disease; CKD, chronic kidney disease; TFA, total saturated fatty acids; MetS, metabolic syndrome.

### Logistic regression of dietary calcium intake and constipation in MetS population

We initially conducted logistic regression analyses to explore the relationship between dietary calcium intake and the incidence of constipation in the MetS population (Table 2). Preliminary results from the unadjusted model demonstrated a significant association (OR: 0.999, 95% CI: 0.998–0.999, *p* < 0.001). Model 1 adjusted for age, gender, marital status, education level, race, smoking habits, alcohol consumption, CVD, CKD, physical activity, and CRP. Even with these adjustments, a significant association between dietary calcium and the risk of constipation was observed (OR: 0.999, 95% CI: 0.999–1.000, *p* = 0.002). Model 2 further adjusted for calcium supplements, energy, protein, total saturated fatty acids, carbohydrate, and fiber based on Model 1, and the association persisted (OR: 0.999, 95% CI: 0.998–1.000, *p* = 0.006). To provide a more intuitive display of the relationship between increased dietary calcium intake and constipation, we standardized the dietary calcium intake data. The results from the standardized data indicated that each standard deviation increase in dietary calcium intake was associated with a 56% decrease in the risk of constipation in the adjusted Model 2 (OR: 0.562, 95% CI: 0.379–0.835, *p* = 0.006). Additionally, a detailed analysis of the relationship between quartiles of dietary calcium intake and the incidence of constipation was conducted. In the unadjusted model, compared to participants in the lowest quartile (Q1), those in Q3 and Q4 of dietary calcium intake showed a significant association with reduced constipation, with ORs of 0.615 (95% CI: 0.393–0.961, *p* = 0.034) and 0.212 (95% CI: 0.107–0.418, *p* < 0.001) respectively. In the adjusted Model 2, the association in Q4 persisted (OR: 0.282, 95% CI: 0.115–0.691, *p* = 0.008) with a trend *p*-value of 0.012.

**Table 2:** Logistic regression analysis of dietary calcium and constipation in the metabolic syndrome population.

Characteristics	Crude model		Model 1		Model 2	
	OR (95% CI)	*p*- value	OR (95% CI)	*p*- value	OR (95% CI)	*p*- value
Dietary Calcium	0.999 (0.998,0.999)	<0.001	0.999 (0.999,1.000)	0.002	0.999 (0.998,1.000)	0.006
Dietary Calcium (standardization)	0.519 (0.388,0.695)	<0.001	0.619 (0.462,0.829)	0.002	0.562 (0.379,0.835)	0.006
Dietary Calcium (categorization)						
Quartile 1	References		References		References	
Quartile 2	0.629 (0.343,1.150)	0.129	0.747 (0.379,1.475)	0.388	0.725 (0.363,1.449)	0.347
Quartile 3	0.615 (0.393,0.961)	0.034	0.803 (0.487,1.322)	0.375	0.767 (0.449,1.309)	0.316
Quartile 4	0.212 (0.107,0.418)	<0.001	0.296 (0.145,0.607)	0.002	0.282 (0.115,0.691)	0.008
*p* for trend		<0.001		0.003		0.012

Dietary Calcium (standardization) indicates that the dietary calcium was standardized so that the mean of the sample becomes 0 and the variance becomes 1. Crude model adjusted for none. Model 1 adjusted for sex, age, ethnicity, marital, smoking, drinking, physical activity, CVD, CKD and CRP. Model 2 further adjusted calcium supplements, energy, protein, total saturated fatty acids, carbohydrate and fiber based on Model 1.

**Figure 2:**
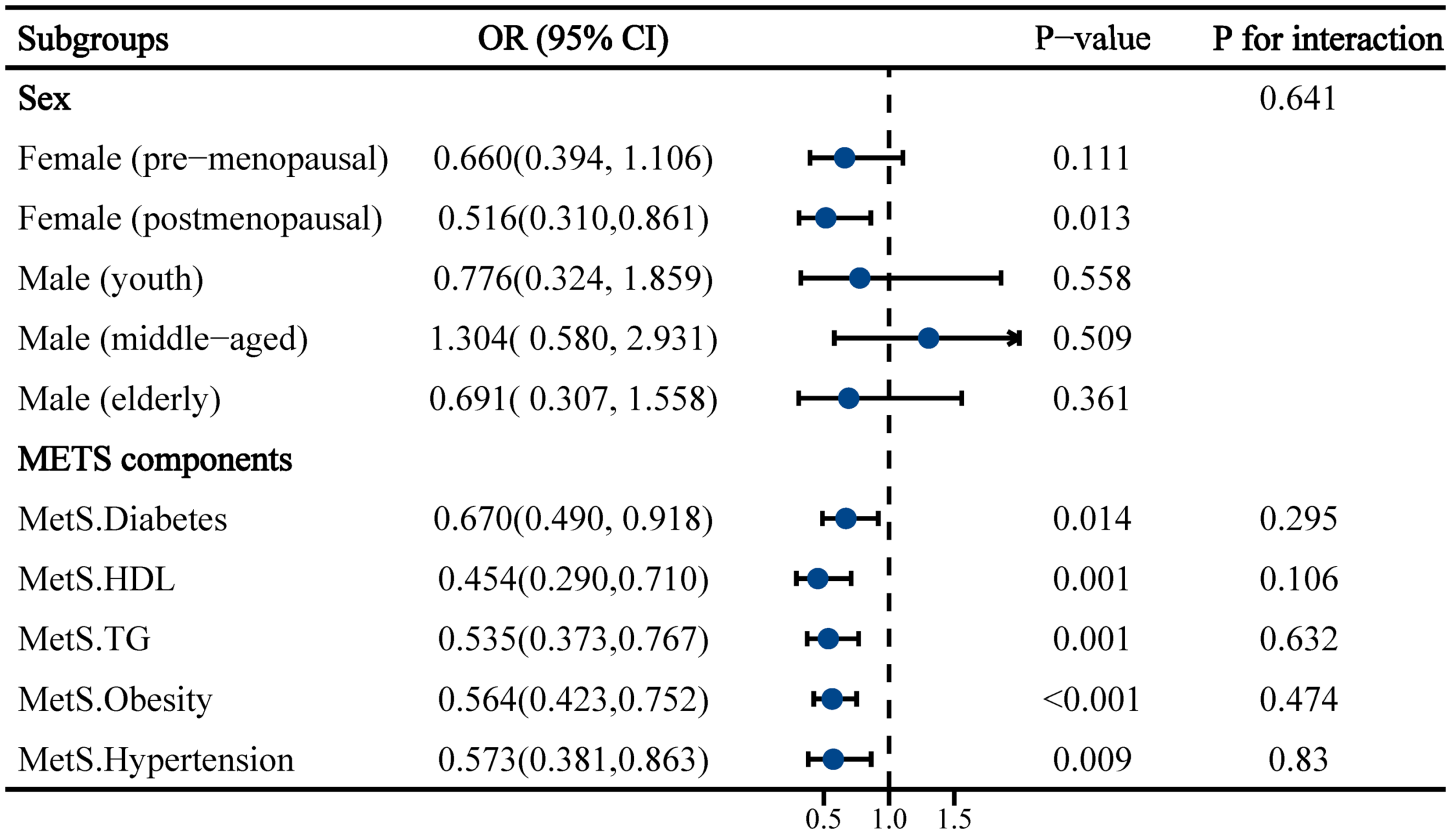
Subgroup analysis of the association between dietary calcium intake and constipation in a population with metabolic syndrome. Adjusted for ethnicity, marital, smoking, drinking, physical activity, CVD, CKD, calcium supplements, energy, protein, carbohydrate and fiber.

Subgroup analyses were subsequently conducted to further elucidate the relationship between increased dietary calcium levels and the risk of constipation in different populations (Figure 2). In the sex subgroup, an association between dietary calcium intake and constipation was only observed in postmenopausal females (OR: 0.516, 95% CI: 0.310 to 0.861, *p* = 0.013). This association was not observed in premenopausal females or in the subgroup of males of all ages. Moreover, significant associations between dietary calcium and constipation were observed across all components of the MetS population.

### Restrictive cubic spline analysis of dietary calcium and constipation

**Figure 3:**
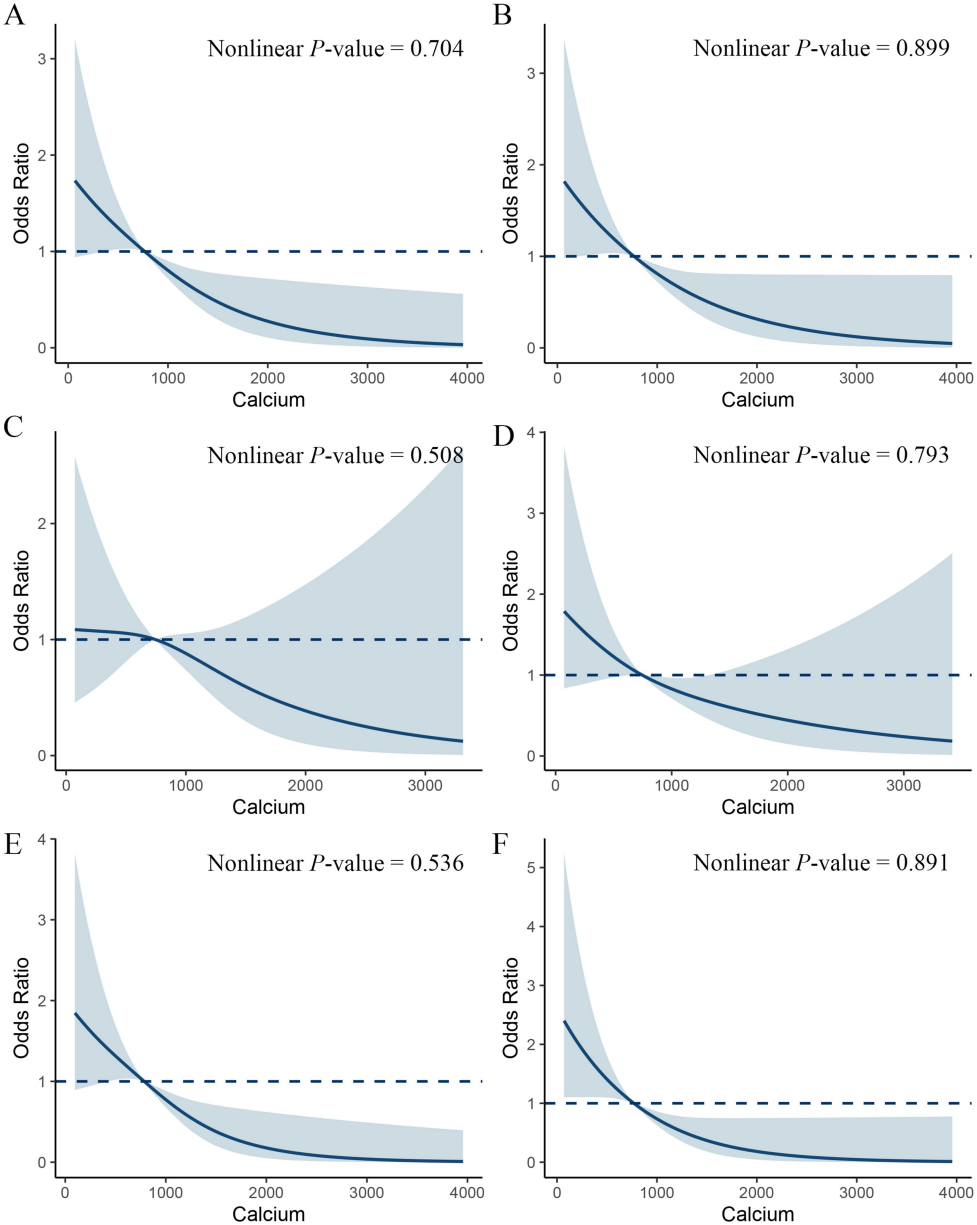
Restricted cubic spline plot of the association between dietary calcium intake and constipation in a metabolic syndrome population. Adjusted for sex, age, ethnicity, marital, smoking, drinking, physical activity, CVD, CKD, calcium supplements, energy, protein, carbohydrate and fiber. **(A)** All MetS populations. **(B)** MetS-obesity **(C)** MetS-hyperglycemia **(D)** MetS-hypertension **(E)** MetS-hypertriglyceridemia **(F)** MetS-Low HDL-C.

To further explore whether there is a nonlinear association between dietary calcium intake and the incidence of constipation among the MetS population, we conducted a restrictive cubic spline analysis (Figure 3). The results indicated that as dietary calcium intake increased, the incidence of constipation significantly decreased, yet no nonlinear relationship was found (non-linearity *p* = 0.704). This association was also observed across all components of MetS.

## Discussion

In this study, the relationship between dietary calcium intake and constipation in patients with MetS, including 4,838 adult participants, was explored using the 2005–2010 NHANES dataset. The results showed a significant linear inverse relationship between dietary calcium intake and the incidence of constipation. Subgroup analyses further emphasized that this effect was more pronounced in postmenopausal women. These results highlight the potential of dietary calcium as a modifiable factor in the prevention of constipation in the MetS population.

The primary characteristic of MetS is abdominal obesity, accompanied by elevated blood pressure, hyperglycemia, and abnormal cholesterol or triglyceride levels. It represents a complex pathophysiological condition that extends beyond traditional metabolic dysfunctions (33). This state significantly increases the risk of cardiovascular diseases, gastrointestinal disorders, and cancers (34). Although there is currently no direct observational evidence linking MetS to constipation risk, studies have shown that MetS is associated with an increased risk of various gastrointestinal diseases, including abdominal pain, irritable bowel syndrome (IBS), and colorectal cancer. Nevertheless, these gastrointestinal diseases and constipation share common pathophysiological mechanisms, primarily involving impaired gastrointestinal motility, dysbiosis of the gut microbiota, and inflammatory responses (35–37). Central obesity, a hallmark of MetS, has been shown to have a significant association with constipation. A study conducted on the Unites States population found that central obesity, as measured by waist-to-height ratio (WHR) and lipid accumulation product (LAP), is strongly correlated with constipation (38). Excessive abdominal fat can compress gastrointestinal organs, slow down gastrointestinal motility, and create obstructions to stool passage (12). Notably, insulin resistance, which is commonly associated with obesity, not only affects glucose and lipid metabolism but also disrupts normal gastrointestinal motility (36). This disruption manifests in conditions such as gastroesophageal reflux disease (GERD), gallstones, and non-alcoholic fatty liver disease (NAFLD), all of which overlap with the etiology of MetS (39, 40). Insulin resistance affects intestinal motility by altering neural control mechanisms and reducing the effectiveness of gastrointestinal hormones that promote motility, resulting in symptoms like constipation (41). Adipose tissue also acts as an endocrine organ, secreting inflammatory substances such as tumor necrosis factor and interleukin-6. These pro-inflammatory mediators alter the composition of the gut barrier and microbiota, further exacerbating gastrointestinal dysfunction (42, 43). Our study demonstrated higher levels of C-reactive protein (CRP) among those with lower dietary calcium intake. A study consistent with our findings showed that visceral obesity in patients with inflammatory bowel disease (IBD) is associated with elevated inflammation levels and a higher prevalence of chronic constipation (44). The inflammatory state increases intestinal permeability, allowing pathogens and toxins easier access to intestinal tissues, thereby aggravating inflammation and causing periodic discomfort and motility issues (45).

Our findings indicate a clear inverse relationship between dietary calcium intake and the incidence of constipation. Although our results do not confirm specific mechanisms, several possible pathways have been proposed. As previously mentioned, the metabolic abnormalities inherent in MetS patients may contribute to digestive tract damage. A dietary calcium intake assessment study among Chinese women suggested that calcium plays a beneficial role in maintaining body composition and preventing abdominal obesity (46). Another study found that in Native American populations, dietary calcium intake was negatively correlated with body mass index and body fat percentage, implying that higher calcium intake may help control weight and reduce body fat, which could indirectly influence constipation incidence (47). The effects of calcium on gastrointestinal health are multifaceted, including enhancing intestinal motility, softening stools, regulating fluid and electrolyte balance, and modulating inflammatory processes. A prospective study conducted in the United Kingdom indicated that dietary calcium intake was inversely related to the incidence of colorectal cancer (48). Calcium can interact with bile acids and fatty acids in the intestine, forming insoluble complexes or soaps, thereby reducing the concentration of free bile acids and lowering the risk of colon cancer (16, 49). This interaction also reduces water reabsorption in the colon, effectively maintaining stool moisture and softness, thus preventing the formation of hard, difficult-to-pass stools (50). Reduced dietary calcium intake has also been associated with IBS, a chronic functional gastrointestinal disorder characterized by abdominal pain or discomfort and changes in bowel habits (18). The pathogenesis of IBS is related to altered gut motility and permeability. Calcium helps maintain the necessary osmotic balance in the intestines by regulating the movement of sodium and potassium across cell membranes, ensuring the appropriate distribution of water (51, 52). Additionally, calcium ions help activate calmodulin, initiating the necessary muscle contractions for effective intestinal motility (53). A double-blind randomized clinical trial demonstrated that calcium and vitamin D supplementation may improve inflammatory markers, such as C-reactive protein and interleukins. Increased dietary calcium intake is often associated with low-inflammatory diets (54). Studies have found significant differences in gut microbiota composition between constipated patients with diets of high inflammatory potential and those following anti-inflammatory diets (55). Given that MetS patients are typically characterized by a chronic low-grade inflammatory state, dietary calcium may help regulate inflammatory pathways within the gut, reducing inflammation-induced changes in gut motility and sensitivity (56, 57).

This study also demonstrated that the association between dietary calcium intake and constipation was more pronounced in postmenopausal women. Research indicates that the effects of calcium on gastrointestinal motility, including the risks of IBS and constipation, differ by gender, with women being two to three times more likely to be affected (58). During menopause, the significant decline in estrogen levels leads to reduced intestinal calcium absorption efficiency. This may result in a decrease in bioavailable calcium necessary for physiological processes, including the maintenance of healthy intestinal motility. One study found that calcium intake in postmenopausal women is inversely correlated with body fat mass (59). This suggests that calcium may reduce the risk of constipation by regulating adipose tissue.

### Limitations

The cross-sectional data from this study limits our ability to establish causality between dietary calcium intake and constipation in the MetS population. Furthermore, the reliance on self-reported data introduces potential biases such as recall bias and social desirability bias, which may affect the accuracy of the reported dietary intakes and health statuses. Additionally, while the NHANES dataset is representative of the United States population, extrapolating these results to populations in other countries with different dietary habits and healthcare systems may not be directly applicable. This limits the generalizability of the findings across diverse global contexts, necessitating cautious interpretation when applying these results outside of the United States.

## Conclusion

This study suggested an association between increased dietary calcium intake and reduced risk of constipation, especially in postmenopausal women.

## Data Availability

The datasets presented in this study can be found in online repositories. The names of the repository/repositories and accession number(s) can be found at: This study is a secondary exploration of the NHANES public database. The data used in the manuscript can be accessed and downloaded from the website https://wwwn.cdc.gov/nchs/nhanes/search/default.aspx.
